# Obesity and Overweight Conditions in Children and Adolescents (6–18 Years) and Their Impact on Craniofacial Morphology: A Systematic Review

**DOI:** 10.3390/children12030377

**Published:** 2025-03-18

**Authors:** Alessio Verdecchia, Carlota Suárez-Fernández, Ivan Menéndez Diaz, Veronica García Sanz, Enrico Spinas, Teresa Cobo

**Affiliations:** 1 Orthodontics Division, Instituto Asturiano de Odontologia, Universidad de Oviedo, 33006 Oviedo, Spain; drmenendez@iaodontologia.com (I.M.D.); dracobo@iaodontologia.com (T.C.); 2 Department of Surgical Sciences, Postgraduate School in Orthodontics, University of Cagliari, 09124 Cagliari, Italy; enricospinas@tiscali.it; 3 Department of Surgery and Medical-Surgical Specialities, School of Medicine and Health Sciences, University of Oviedo, 33003 Oviedo, Spain; suarezcarlota@uniovi.es; 4 Orthodontics Teaching Unit, Department of Stomatology, Faculty of Medicine and Dentistry, University of Valencia, 46010 Valencia, Spain; veronica.garcia-sanz@uv.es

**Keywords:** childhood obesity, craniofacial morphology, skeletal maturation, orthodontics treatment

## Abstract

**Background**: Childhood obesity and overweight conditions impact systemic health and craniofacial development. **Objectives**: This review assessed the influence of elevated body mass index (BMI) on craniofacial morphology, considering age, sex, and ethnicity. **Methods**: A comprehensive search of Scopus, Web of Science, Embase, Cochrane, PubMed, and OpenGrey was conducted following PRISMA guidelines. Ten cross-sectional studies involving 1383 individuals aged 6 to 18 years were included. The sample comprised 812 females and 571 males, with most studies focusing on adolescents aged 12–18 years of different ethnicities depending on the study. Craniofacial structures were compared between overweight/obese and normal weight groups through cephalometric analysis. Study quality was assessed using the Newcastle–Ottawa Scale (NOS). **Results**: Overweight and obese individuals showed significant craniofacial changes, including increased anterior cranial base length, maxillary and mandibular dimensions, bimaxillary prognathism, and greater soft tissue thickness. These alterations may be influenced by differences in tissue composition, hormonal fluctuations, fat-to-bone ratio, and metabolic disorders. Variations in skeletal divergence, dental alignment, and airway space were also observed. The methodological quality ranged from moderate to high. **Conclusions**: Excess weight during growth is linked to distinct craniofacial alterations. Orthodontic diagnostics should integrate metabolic and hormonal considerations to optimize treatment outcomes. These changes should be carefully considered by orthodontists and pediatric dentists. Longitudinal studies are needed to understand the long-term effects of obesity on craniofacial development.

## 1. Introduction

Childhood obesity and overweight conditions have emerged as critical global health concerns, influencing not only systemic health but also the growth and development of craniofacial structures. Defined by excessive fat accumulation that may impair health, these conditions are the result of a multifaceted interplay of genetic, environmental, and behavioural factors [1,2]. Epidemiological studies highlight the alarming rise in pediatric obesity worldwide, with significant variations across demographics and socioeconomic groups [3].

The impact of obesity on craniofacial development is evident in its association with advanced skeletal and dental maturation. Obese children often experience early dental eruption and advanced bone age, phenomena that have been linked to hormonal alterations and inflammatory processes [4,5]. For example, elevated leptin levels in obesity accelerate skeletal and dental development, while pro-inflammatory adipokines further exacerbate these changes [6,7]. Additionally, obesity-induced changes in bone metabolism can compromise bone quality despite increased bone mass, leading to higher fracture risks [8,9].

From a dental perspective, obesity has been associated with an increased prevalence of malocclusion and early dental eruption [10,11]. Obese children often exhibit more erupted teeth than their non-obese peers, potentially leading to malocclusions and greater susceptibility to caries [12,13]. These findings underscore the need for early orthodontic and dental evaluations to mitigate long-term complications.

The systemic effects of obesity also extend to orthodontic treatment outcomes. Studies suggest that while obesity may not significantly affect treatment duration, it influences tooth movement dynamics due to altered inflammatory and hormonal responses [14,15]. Moreover, periodontal challenges such as increased plaque accumulation and inflammation are more pronounced in obese patients, highlighting the complexity of managing orthodontic care in this population [16].

The multifactorial etiology of obesity involves poor dietary habits, reduced physical activity, and genetic predispositions, all of which contribute to its growing prevalence and associated complications [17,18]. These factors disrupt normal growth patterns, leading to early puberty onset in obese boys and altered growth trajectories [19]. Importantly, the interplay of systemic and craniofacial changes in obese children necessitates integrated healthcare approaches to address both nutritional and orthodontic challenges effectively [20].

The objective of this systematic review is to evaluate the available evidence on the impact of obesity and overweight conditions during growth on craniofacial morphology. By examining the clinical implications and potential interventions, this review seeks to provide a comprehensive understanding of how excess weight affects the development and structure of craniofacial features.

## 2. Materials and Methods

### 2.1. Information Sources and Search Strategy

The systematic review adhered to the Preferred Reporting Items for Systematic Reviews and Meta-Analyses (PRISMA) guidelines [21]. The protocol was registered in the Prospective Register of Systematic Reviews (PROSPERO) on 30 November 2024 (registration number CRD42024620599). The primary research question was “How do obesity and overweight impact the development of craniofacial structures in growing patients?” A comprehensive search was conducted on 24 December 2024 across Scopus, Web of Science, Embase, Cochrane, and PubMed databases. Additionally, grey literature was explored through OpenGrey. The research was conducted without the application of temporal or language filters to ensure the retrieval of all available scientific information. The detailed search strategy is presented in Table 1.

### 2.2. Eligibility Criteria

Studies were selected based on the following criteria, structured according to the PICO framework:

- Population (P): Children and adolescents aged 6 to 18 years with obesity or overweight conditions.
- Intervention (I): No intervention applied.
- Comparison (C): Normal weight children and adolescents within the same age range.
- Outcome (O): Differences in the development of craniofacial structures among growing individuals with obesity or overweight conditions.

The inclusion criteria for this study encompassed original research studies, including clinical trials, case–control studies, cohort studies, cross-sectional studies, longitudinal studies, prospective studies, and retrospective studies. Eligible participants were required to be between 6 and 18 years of age. Additionally, studies had to perform cephalometric analysis to assess craniofacial development.

Exclusion criteria included studies involving subjects with syndromes, congenital anomalies, or systemic conditions that could affect craniofacial development. Publications such as case reports, case series, reviews, systematic reviews, and meta-analyses were not considered. Furthermore, studies including participants younger than 6 years or older than 18 years were excluded, as well as those lacking skeletal cephalometric measurements or relevant data.

### 2.3. Data Extraction and Synthesis

Two authors (A.V. and C.S.F.) independently conducted the research process and subsequently screened the obtained results. To evaluate the agreement level among the reviewers, Cohen’s kappa coefficient [22] was calculated. In case of disagreement, a third reviewer (T.C.) was consulted. The authors demonstrated a substantial agreement (Cohen’s kappa: 0.66). For each study included in the research, we collected the following parameters: publication information (authors of the study and year of publication), country where the study was conducted, study design (type of study), sample characteristics (including age, sex, ethnicity, size, and number of subjects classified by BMI categories: obese (OB), overweight (OW), normal weight (NW), and underweight (UW)), dental maturation assessment, skeletal maturation evaluation, craniofacial morphology assessment, and body mass assessment.

Furthermore, we investigated the morphological differences in craniofacial structures between individuals who were obese (OB) and overweight (OW) compared to those with normal weight (NW), emphasizing significant cephalometric disparities between the elevated BMI groups (OB, OW) and the control group comprising NW subjects.

### 2.4. Quality Assessment

A thorough quality evaluation of the studies was performed using the Newcastle–Ottawa scale (NOS) [23] for cross-sectional studies.

The Newcastle–Ottawa scale assesses the methodological quality of cross-sectional studies by evaluating three main criteria, namely selection, comparability, and outcome, assigning up to 3 points per section for a maximum total of 9 points. Studies with a score ≥ 8 was considered high quality, a score between 4 and 7 was considered as moderate quality, and a score ≤ 3 was considered as low-quality.

This scale was utilized to perform the risk of bias assessment.

## 3. Results

The search strategy yielded a total of 1792 publications distributed across the following databases: EMBASE (866), PubMed (445), Web of Science (152), Scopus (168), and Cochrane (161). No articles were identified through manual searches or grey literature sources, such as OpenGrey. After removing duplicates, 1586 unique records remained.

Following a preliminary screening of titles and abstracts, 1549 articles were excluded for not meeting the inclusion criteria. The full text of the remaining 37 articles was assessed for eligibility. Of these, 27 articles were excluded for the following reasons: 5 focused exclusively on skeletal maturation, 17 addressed only dental maturation, and 5 discussed both skeletal and dental maturation but without analyzing morphological changes in craniofacial structures.

**Figure 1:**
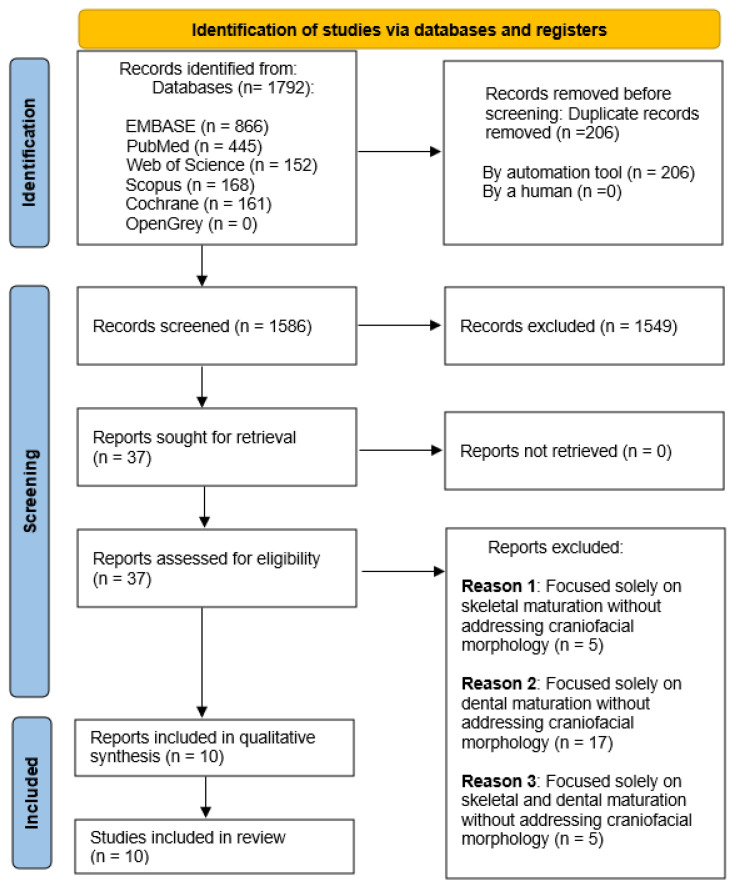
PRISMA flowchart; flow diagram of the performed search.

Ultimately, 10 studies were deemed eligible and included in the qualitative analysis. The complete details of the search strategy and selection process are illustrated in the flow chart presented in Figure 1.

### 3.1. Description of the Included Studies

Table 2 describes the main characteristics of the studies included of this systematic review.

#### 3.1.1. Country and Study Design

This systematic review includes studies published between 2002 and 2024 from various countries, with the largest representation coming from the United States, with four studies [28,29,31,32]. This is followed by two studies each from Turkey [27,30] and Sweden [24,25], and one study each from Italy [26] and Spain [33].

All of the included studies employed cross-sectional designs using normal weight individuals as the control group [24,25,26,27,28,29,30,31,32,33].

#### 3.1.2. Sample Characteristics

The studies included in this review feature sample sizes ranging from 48 participants to as many as 400. In total, the pooled sample size across all studies amounts to 1383 participants, with a relatively balanced distribution between females (812 participants) and males (571 participants).

Participants ranged in age from 5 to 19 years, with most studies focusing on adolescents between 12 and 18 years of age. For example, Karaman A et al. [30] included participants aged 14.0 to 18.0 years, while Danze A et al. [28] examined a younger cohort aged 5.0 to 10.0 years.

Regarding ethnicity, only two studies explicitly reported that the analyzed sample consisted entirely of Caucasian populations [26,33]. However, studies conducted in the United States included more diverse populations, encompassing African American, Caucasian, Asian, Hispanic, and other groups [28,29,32].

Notably, five studies did not specify the ethnicity of the participants, which limits the generalizability of their findings across different populations [24,25,27,30,31].

#### 3.1.3. Body Mass Categories

The participants of the included studies were categorized based on their body mass index (BMI) into four groups, namely obese, overweight, normal weight, and underweight. Four studies exclusively included obese patients without mentioning the presence of overweight individuals [24,25,26,31]. Another four studies made a clear distinction between obese and overweight patients, including them as separate groups [27,29,30,33]. In contrast, two studies differentiated between obese and overweight participants but combined overweight individuals within the obese group without providing specific numerical data for the overweight category [28,32]. No underweight patients were included [24,25,26,27,28,29,30,31,32,33].

#### 3.1.4. Craniofacial Morphology and Maturation Parameters

To assess craniofacial morphology, all the studies utilized cephalometric tracings to determine linear and angular measurements, employing various types of cephalometric reference points [24,25,26,27,28,29,30,31,32,33].

Additionally, four studies [28,31,32,33] reported complementary diagnostic parameters related to skeletal vertebral maturation and dental maturation [34,35].

#### 3.1.5. Body Mass Assessment

All included studies used BMI-for-age growth charts, either from the CDC or the WHO [36,37], to classify participants into their respective weight categories [24,25,26,27,28,29,30,31,32,33].

### 3.2. Results of Cephalometric Analysis

As illustrated in Table 3, all cephalometric values where statistically significant changes (*p* < 0.05) were reported between subjects with obesity and overweight compared to normal weight individuals have been described.

#### 3.2.1. Maxillofacial Length Parameters

A marked enlargement was observed in the anterior cranial base length (S-N) across several studies [24,25,26,30], highlighting a significant cranial extension in obese and overweight groups compared to normal weight counterparts. The maxillary length demonstrated consistent elongation, evidenced in parameters such as (Pm-A) [24,25,26], (Co-A) [30], and (PNS-A) [31].

Mandibular length, encompassing measurements like (Cd-Pgn), (Ar-Gn), and (Co-Gn), also showed notable growth across various studies [24,25,28,29,30,31]. Similarly, the corpus length (Go-Pg) and posterior facial height (S-Go) exhibited significant increments.

In contrast, the anterior facial height (N-Me) displayed divergent results, with studies reporting both increases [28,30,32] and a decrease in one study [31]. The upper anterior facial height (Na-Sp) notably decreased [24], whereas parameters like lower anterior facial height (ANS-Gn) and maxillary dentolabial height (ANS-Pr) predominantly showed upward trends. Additionally, dimensions such as ramus length, mandibular corpus height, facial centroid size, mandibular centroid size, and vertical skeletal dimension (PP/GoMe) were consistently larger in individuals with a higher BMI [25,29,30].

#### 3.2.2. Jaw Projection and Prognathism Parameters

Maxillary projection (SNA, NPerp-A) and mandibular projection (SNB, NPerp-Pg) consistently exhibited increased prominence in subjects with elevated BMI [25,28,30,32,33]. Similarly, pogonion projection (SNPg) showed significant augmentation across multiple studies [25,28,32].

Both maxillary prognathism (S-Na-Ss) and mandibular prognathism (S-Na-Sm) were reported as significantly increased in obese individuals compared to their normal weight peers [24,29]. Additionally, mandibular alveolar prognathism (ML/CL) presented higher measurements [25].

#### 3.2.3. Skeletal Divergence Parameters

An accentuated mandibular growth direction (Ar-Go-Gn) and a more pronounced clockwise cranial base rotation (N-S-Ba) were observed, particularly in obese subjects [31]. Reductions were noted in the intermaxillary plane angle (NL/ML) [26] and the maxillary plane angle (NL/SN) [25], while the jaw angle (RL/ML) demonstrated increased dimensions [24].

#### 3.2.4. Dental Positions

Dental inclination patterns revealed an upward shift in both upper incisor inclination (U1/NL) and lower incisor inclination (L1/ML) in relation to BMI variations [25]. However, other studies [24,26,27,28,29,30,31,32,33] did not reveal significant cephalometric alterations concerning dental positioning.

#### 3.2.5. Facial Soft Tissue Thickness

Soft tissue measurements, including nasion (N-N’), glabella (G-G’), pogonion (Pg-Pg’), gnathion (Gn-Gn’), rhinion (Rhi-Rhi’), subnasale (ANS-Sn), labiale superius (Ls), stomion (Sto), labiale inferius (Li), and labiomentale (B-B’), indicated considerable thickening in obese individuals [27,30]. The facial soft tissue profile (convexity) tended towards a straighter contour in the obese cohort [25].

#### 3.2.6. Airway Analysis

A substantial expansion in the nasopharyngeal airway (Pm-Ad2) was identified [24], suggesting morphological adaptations of the upper airway in response to craniofacial structural modifications associated with increased BMI.

Regarding gender differences, all studies [24,25,26,27,28,30,31,32,33] reported significant morphological cephalometric changes in both sexes within the obese and overweight groups compared to the normal weight group, with the exception of the study by Gordon LA et al. [29]. In this study, a notable increase in mandibular length, maxillary prognathism, and mandibular projection (SNB) was observed exclusively in obese and overweight females compared to their normal weight counterparts, while no significant differences were detected in the male group.

### 3.3. Risk of Bias Assessment

The complete NOS scores for each study can be viewed in Table 4, which highlights their respective ratings across the domains of selection, comparability, and outcome.

Based on this assessment, four studies [28,29,31,32] were classified as high quality, indicating robust methodological rigour with minimal risk of bias. These studies demonstrated strong performance across all domains, particularly in controlling for confounding factors and utilizing appropriate statistical analyses.

The majority of studies, totaling six [24,25,26,27,30,33], fell into the moderate-quality category, reflecting acceptable methodological quality but with some potential sources of bias. These studies often lacked detailed reporting on non-respondents and showed variability in controlling for confounding variables, which slightly affected their overall quality ratings.

No studies included in this review were classified as low quality, indicating that all studies met a minimum standard of methodological rigour. Table 4 provides a comprehensive overview of the NOS scores, allowing for an objective comparison of the methodological quality across the included studies.

## 4. Discussion

This systematic review elucidates the significant craniofacial skeletal and soft tissue modifications observed in overweight and obese individuals compared to their normal weight counterparts. The findings reveal substantial increases in anterior cranial base length, maxillary and mandibular dimensions, prognathism, and soft tissue thickness. These observations corroborate the existing literature, reinforcing the premise that obesity exerts a measurable influence on craniofacial morphology and orthodontic biomechanics.

A pronounced elongation of the anterior cranial base (S-N) was documented across multiple studies, suggesting an obesity-associated cranial extension [24,25,26,30,31]. These findings align with research linking obesity-related hormonal fluctuations, particularly leptin and insulin-like growth factor-1 (IGF-1), to skeletal growth regulation [38,39]. Similarly, mandibular length measurements (Cd-Pgn, Ar-Gn, Co-Gn) exhibited significant increases [24,25,28,29,30,31]. This is further supported by Luo et al. [40], who demonstrated an accelerated alveolar bone remodelling process in obese individuals, while Damanaki et al. [41] identified a notable increase in alveolar crest resorption, potentially contributing to these morphological alterations.

Maxillary (SNA, NPerp-A) and mandibular (SNB, NPerp-Pg) projections were significantly more pronounced in obese cohorts [25,28,30,32,33]. These findings parallel Balof-Tuncer et al. [42], who demonstrated that obesity-related endocrine disruptions contribute to increased facial convexity, augmented mandibular plane angles, and altered maxillary development. Moreover, Zhao et al. [43] underscored the role of metabolic disturbances in midpalatal suture remodelling during rapid maxillary expansion (RME), suggesting that obesity-associated FTO protein enhances osteogenic differentiation in suture mesenchymal stem cells, potentially influencing maxillary expansion outcomes.

Dental and skeletal maturation were observed to advance more rapidly in obese pediatric populations, indicating accelerated craniofacial growth trajectories [5]. Some studies identified an increased inclination of upper and lower incisors in individuals with elevated BMI [25], whereas others reported negligible cephalometric alterations in dental positioning [24,26,27,28,29,30,31,32,33]. Nicholas et al. [44] emphasized that obesity is strongly associated with accelerated dental eruption, likely mediated by systemic metabolic factors, a finding corroborated by Mohamedhussein et al. [45], who linked adipokine activity to precocious molar eruption.

Thongudomporn et al. [46] explored the impact of maximum bite force (MBF) on alveolar bone morphology, revealing its moderate influence on alveolar thickness and configuration without substantial effects on arch width. Notable sex differences were observed, with males exhibiting greater alveolar dimensions. These insights are further reinforced by Castelo et al. [47], who highlighted the impact of obesity on masticatory efficiency, and Conith et al. [48], who demonstrated that craniofacial bone adaptation is significantly modulated by muscle-induced mechanical loading.

Obesity was also associated with notable soft tissue modifications, particularly increased thickness in the nasion, glabella, pogonion, and gnathion regions. These findings align with those of Luo et al. and Thongudomporn et al. [40,46]. Furthermore, Luo et al. [40] emphasized that increased adiposity alters soft tissue distribution, a consideration that should be integrated into orthodontic diagnostic and treatment planning.

Airway adaptations were evident, with obese individuals exhibiting a significant increase in nasopharyngeal airway dimensions (Pm-Ad2) [24]. Huang et al. [49] posited that obesity exerts a greater influence on airway obstruction than skeletal morphology, reinforcing the necessity of a comprehensive orthodontic and respiratory assessment in affected individuals.

The interplay between obesity and bone metabolism may further complicate orthodontic treatment. Ruiz-Heiland et al. [50] investigated leptin’s effects on cementoblasts during orthodontic tooth movement, revealing that leptin induces an inflammatory response, increases the apoptosis of cementoblasts, and promotes PGE2 release, which could modify periodontal responses and orthodontic stability. Additionally, Fudalej et al. [51] critically evaluated the cervical vertebral maturation (CVM) method, suggesting that its predictive reliability is compromised in obese individuals due to altered skeletal growth dynamics, advocating for a more individualized approach to orthodontic assessment.

Obesity has also been associated with expedited orthodontic tooth movement (OTM), attributed to heightened pro-inflammatory cytokines and adipokines [14,40]. However, Consolaro et al. [15] contended that obesity does not exert a significant impact on orthodontic movement velocity, emphasizing the need for further empirical validation. Additionally, periodontal considerations remain pertinent, as Li et al. [52] reported an association between elevated BMI and increased periodontal inflammation, which may impact long-term orthodontic stability. Neeley et al. [53] further underscored the need for tailored orthodontic protocols given that obese patients experience earlier pubertal growth spurts and altered bone metabolism.

The findings of this systematic review underscore the multifaceted influence of obesity on craniofacial morphology, orthodontic treatment considerations, and airway function.

Despite the inclusion of studies with moderate-to-high methodological quality, certain limitations must be acknowledged as follows:

- The majority of included studies are cross-sectional, which limits the ability to establish causal relationships.
- Many studies do not account for racial and ethnic variations in craniofacial morphology, potentially influencing generalizability.
- The wide age range considered in some studies may introduce developmental variability, affecting the interpretation of growth patterns.
- Specific hormonal analyses to determine the precise physiopathological etiology of craniofacial changes were not conducted.
- There is a lack of long-term prospective studies, which are essential for understanding the sustained effects of obesity on craniofacial development and orthodontic outcomes. Given the rising prevalence of childhood obesity, orthodontic diagnostics should integrate metabolic and hormonal considerations to optimize treatment outcomes. Future investigations should focus on longitudinal studies assessing the enduring effects of obesity on craniofacial development, along with further elucidation of the molecular pathways linking adipokine activity to orthodontic and skeletal dynamics.

## 5. Conclusions

This systematic review highlights the significant influence of obesity on craniofacial morphology. Based on the current evidence, the following key points can be drawn:

- Obesity is associated with increased craniofacial dimensions, including maxillary and mandibular length, as well as increased jaw projections and jaw prognathism.
- Obese and overweight individuals exhibit greater facial hyperdivergence.
- Soft tissue thickness is significantly greater in obese individuals.
- Despite these findings, further longitudinal and prospective studies are necessary to establish causal relationships and refine clinical guidelines for orthodontic treatment in obese patients.

The association with obesity leads to an accelerated dental and skeletal age. Pediatric dentists and orthodontists should consider that this acceleration impacts craniofacial morphology, necessitating early interception to optimize the timing of dentofacial orthopedic treatment in growing patients. This aspect should be thoroughly documented in the patient’s medical history to ensure appropriate clinical management.

## Figures and Tables

**Table 1:** Search strategy for each database.

Database	Search Strategy
Scopus	(TITLE-ABS-KEY (“obesity”) OR TITLE-ABS-KEY (“childhood obesity”) OR TITLE-ABS-KEY (“BMI”) OR TITLE-ABS-KEY (“overweight”) AND TITLE-ABS-KEY (“dental eruption”) OR TITLE-ABS-KEY (“facial growth”) OR TITLE-ABS-KEY (“cervical vertebral maturation”) OR TITLE-ABS-KEY (“skull growth”) OR TITLE-ABS-KEY (“dental maturation”) OR TITLE-ABS-KEY (“orthodontics”))
Web of Science	ALL = ((“obesity” OR “childhood obesity” OR “BMI” OR “overweight”) AND (“dental eruption” OR “facial growth” OR “cervical vertebral maturation” OR “skull growth” OR “dental maturation” OR “orthodontics”))
Embase	(obesity:ti,ab,kw OR ‘childhood obesity’:ti,ab,kw OR ‘body mass’:ti,ab,kw OR ‘overweight’:ti,ab,kw) AND ‘tooth eruption’:ti,ab,kw OR ‘face growth’:ti,ab,kw OR ‘skull development’:ti,ab,kw OR ‘dental maturation’:ti,ab,kw OR ‘cervical vertebral maturation’:ti,ab,kw)
Cochrane	(“obesity”):ti,ab,kw OR (“BMI”):ti,ab,kw AND (“facial growth”):ti,ab,kw OR (“cervical vertebral maturation”):ti,ab,kw OR (“dental eruption”):ti,ab,kw
Pubmed	(“obesity” [All Fields] OR “childhood obesity” [All Fields] OR “BMI” [All Fields] OR “overweight” [All Fields]) AND (“dental eruption” [All Fields] OR “facial growth” [All Fields] OR “cervical vertebral maturation” [All Fields] OR “skull growth” [All Fields] OR “dental maturation” [All Fields] OR “orthodontics” [All Fields])

**Table 2:** The main characteristics of included studies.

Author/Year (Country)	Age/Sex/Ethnicity	Sample Size	OB (N)	OW (N)	NW (N)	UW (N)	Dental and Skeletal Maturation Assessment
Ohrn K et al., 2002 (Sweden) [24]	F: 15.7 ± 0.82 y M: 14.6 ± 0.53 y 25 F 14 M control group: 25 F 14 M NR	78	39	NR	39	NR	NR
Sadeghianrizi A et al., 2005 (Sweden) [25]	F: 15.6 ± 0.83 y M: 13.9 ± 0.98 y 27 F 23 M NR	100	50	NR	50	NR	NR
Giuca MR et al., 2013 (Italy) [26]	9.8 ± 2.1 y Control group: 9.9 ± 2.5 y 22 F 28 M Caucasian	50	25	NR	25	NR	NR
Buyuk SK et al., 2019 (Turkey) [27]	12.92 to 17.53 y 50 F 30 M NR	80	15	18	47	NR	NR
Danze A et al., 2021 (United States) [28]	5.0 to 10.0 y 223 F 177 M African American: 43 Caucasian: 243 Asian: 16 Other: 9	400	OB + OW 200		200	NR	- Demerjian - CVM
Gordon LA et al., 2021 (United States) [29]	9.0 to 19.0 y 95 F 86 M African American: 20 Asian: 9 Caucasian: 134 Multiracial: 3 Unknown: 15	181	47	35	93	6	NR
Karaman A et al., 2021 (Turkey) [30]	14.0 to 18.0 y 15.65 ± 1.16 y 135 F 157 M NR	292	95	93	104	NR	NR
Vora SR et al., 2022 (United States) [31]	7.6 to 16.4 y 16 F 32 M NR	48	24	NR	24	NR	- Demerjian - CVM
Hancock S et al., 2024 (United States) [32]	8 to 14 y 183 F 143 M Control group: 86 F, 72 M African American 34 Caucasian 192 Hispanic 82 Others 19	326	168 OB + OW without distinction	168 OB + OW without distinction	158	NR	- NR - CVM
Verdecchia A. et al., 2024 (Spain) [33]	11.96 ± 2.44 y 94 F 84 M Caucasian	178	26	37	115	NR	- Demerjian - CVM

Abbreviations: F, female; M, male; y, years; NR, not reported; OB, obesity; OW, overweight; NW, normal weight; UW, underweight; CVM, cervical vertebral maturation.

**Table 3:** Morphological differences in craniofacial structures between obese/overweight patients and normal weight patients.

Craniofacial Parameters	Significant Cephalometric Differences (OB, OW vs. NW)—*p* < 0.05		Authors/Year
**Maxillofacial Length Parameters**	Anterior Cranial Base Length (S-N)	Increased	Ohrn K et al., 2002 [24]; Sadeghianrizi A et al., 2005 [25]; Giuca MR et al., 2013 [26]; Karaman A et al., 2021 [30]
	Maxillary Length (Pm-A)	Increased	Ohrn K et al., 2002 [24]; Sadeghianrizi A et al., 2005 [25]; Giuca MR et al., 2013 [26]
	Maxillary Length (Co-A)	Increased	Karaman A et al., 2021 [30]
	Maxillary Length (PNS-A)	Increased	Vora SR et al., 2022 [31]
	Mandibular Length (Cd-Pgn)	Increased	Ohrn K et al., 2002 [24]; Sadeghianrizi A et al., 2005 [25]; Gordon LA et al., 2021 [29]
	Mandibular Length (Ar-Gn)	Increased	Danze A et al., 2021 [28]
	Mandibular Length (Co-Gn)	Increased	Karaman A et al., 2021 [30]; Vora SR et al., 2022 [31]
	Corpus Length (Go-Pg)	Increased	Ohrn K et al., 2002 [24]; Sadeghianrizi A et al., 2005 [25]
	Posterior Facial Height (S-Go)	Increased	Ohrn K et al., 2002 [24]; Sadeghianrizi A et al., 2005 [25]; Danze A et al., 2021 [28]; Karaman A et al., 2021 [30]
	Anterior Facial Height (N-Me)	Increased/Decreased	Danze A et al., 2021 [28]; Karaman A et al., 2021 [30]; Hancock S et al., 2024 [32]; Vora SR et al., 2022 [31]
	Upper Anterior Facial Height (Na-Sp)	Decreased	Ohrn K et al., 2002 [24]
	Lower Anterior Facial Height (ANS-Gn)	Increased	Sadeghianrizi A et al., 2005 [25]
	Maxillary Dentolabial Height (ANS-Pr)	Increased	Sadeghianrizi A et al., 2005 [25]
	Ramus Length	Increased	Gordon LA et al., 2021 [29]
	Mandibular Corpus Height	Increased	Gordon LA et al., 2021 [29]
	Facial Centroid Size	Increased	Gordon LA et al., 2021 [29]
	Mandibular Centroid Size	Increased	Gordon LA et al., 2021 [29]
	Vertical Skeletal Dimension (PP/GoMe)	Increased	Karaman A et al., 2021 [30]
**Jaws Projection and Prognathism Parameters**	Maxillary Projection (SNA, NPerp-A)	Increased	Sadeghianrizi A et al., 2005 [25]; Danze A et al., 2021 [28]; Karaman A et al., 2021 [30]; Hancock S et al., 2024 [32]; Verdecchia A et al., 2024 [33]
	Mandibular Projection (SNB, NPerp-Pg)	Increased	Sadeghianrizi A et al., 2005 [25]; Danze A et al., 2021 [28]; Gordon LA et al., 2021 [29]; Karaman A et al., 2021 [30]; Hancock S et al., 2024 [32]; Verdecchia A et al., 2024 [33]
	Pogonion Projection (SNPg)	Increased	Sadeghianrizi A et al., 2005 [25]; Danze A et al., 2021 [28]; Hancock S et al., 2024 [32]
	Maxillary Prognathism (S-Na-Ss)	Increased	Ohrn K et al., 2002 [24]; Gordon LA et al., 2021 [29]
	Mandibular Prognathism (S-Na-Sm)	Increased	Ohrn K et al., 2002 [24]
	Mandibular Alveolar Prognathism (ML/CL)	Increased	Sadeghianrizi A et al., 2005 [25]
**Facial Soft Tissue Thickness**	Nasion (N-N’)	Increased	Buyuk SK et al., 2019 [27]; Karaman A et al., 2021 [30]
	Glabella (G-G’)	Increased	Buyuk SK et al., 2019 [27]; Karaman A et al., 2021 [30]
	Pogonion (Pg-Pg’)	Increased	Buyuk SK et al., 2019 [27]; Karaman A et al., 2021 [30]
	Gnathion (Gn-Gn’)	Increased	Buyuk SK et al., 2019 [27]; Karaman A et al., 2021 [30]
	Rhinion (Rhi-Rhi’)	Increased	Karaman A et al., 2021 [30]
	Subnasale (ANS-Sn)	Increased	Karaman A et al., 2021 [30]
	Labiale Superius (Ls)	Increased	Karaman A et al., 2021 [30]
	Stomion (Sto)	Increased	Karaman A et al., 2021 [30]
	Labiale Inferius (Li)	Increased	Karaman A et al., 2021 [30]
	Labiomentale (B-B’)	Increased	Karaman A et al., 2021 [30]
	Chin Prominence	Increased	Gordon LA et al., 2021 [29]; Vora SR et al., 2022 [31]
	Facial Soft Tissue Profile (Convexity)	More straight profiles	Sadeghianrizi A et al., 2005 [25]
**Skeletal Divergence**	Mandibular Growth Direction (Ar-Go-Gn)	Increased	Vora SR et al., 2022 [31]
	Cranial Base Rotation (N-S-Ba)	More clockwise rotation	Vora SR et al., 2022 [31]
	Intermaxillary Plane Angle (NL/ML)	Decreased	Giuca MR et al., 2013 [26]
	Jaw Angle (RL/ML)	Increased	Ohrn K et al., 2002 [24]
	Maxillary Plane Angle (NL/SN)	Decreased	Sadeghianrizi A et al., 2005 [25]
**Dental Positions**	Upper Incisor Inclination (U1/NL)	Increased	Sadeghianrizi A et al., 2005 [25]
	Lower Incisor Inclination (L1/ML)	Increased	Ohrn K et al., 2002 [24]
**Airway Analysis**	Nasopharyngeal Airway (Pm-Ad2)	Increased	Ohrn K et al., 2002 [24]

Abbreviations: OB, obesity; OW, overweight; NW, normal weight; S-N, sella–nasion; Pm-A, pronasale to point A; Co-A, condylion to point A; PNS-A, posterior nasal spine to point A; Cd-Pgn, condylion to pogonion; Ar-Gn, articulare to gnathion; Co-Gn, condylion to gnathion; Go-Pg, gonion to pogonion; S-Go, sella to gonion; N-Me, nasion to menton; Na-Sp, nasion to spina nasalis; ANS-Gn, anterior nasal spine to gnathion; ANS-Pr, anterior nasal spine to pronasale; PP/GoMe, palatal plane to gonion–menton plane; SNA, sella–nasion–point A angle; NPerp-A, nasion perpendicular to point A; SNB, sella–nasion–point B angle; NPerp-Pg, nasion perpendicular to pogonion; SNPg, sella–nasion to pogonion angle; S-Na-Ss, sella–nasion–subspinale angle; S-Na-Sm, sella–nasion–supramentale angle; ML/CL, mandibular line to cranial line; N-N’, nasion to soft tissue nasion; G-G’, glabella to soft tissue glabella; Pg-Pg’, pogonion to soft tissue pogonion; Gn-Gn’, gnathion to soft tissue gnathion; Rhi-Rhi’, rhinion to soft tissue rhinion; ANS-Sn, anterior nasal spine to subnasale; Ls, labiale superius; Sto, stomion; Li, labiale inferius; B-B’, labiomentale to soft tissue labiomentale; Ar-Go-Gn, articulare–gonion–gnathion angle; N-S-Ba, nasion–sella–basion angle; NL/ML, nasal line to mandibular line; RL/ML, ramus line to mandibular line; NL/SN, nasal line to sella–nasion line; U1/NL, upper incisor to nasal line; L1/ML, lower incisor to mandibular line; Pm-Ad2, pronasale to adenoid point 2.

**Table 4:** Risk of bias assessment for all the selected studies for systematic review.

Author/Year	Selection	Comparability	Outcome	Quality Score (Max 9)
Öhrn K et al., 2002 [24]	3	1	2	6
Sadeghianrizi A et al., 2005 [25]	3	2	2	7
Giuca M.R. et al., 2013 [26]	3	1	2	6
Buyuk S.K. et al., 2018 [27]	3	1	2	6
Danze A. et al., 2020 [28]	4	2	2	8
Gordon L.A. et al., 2021 [29]	4	2	2	8
Karaman A. et al., 2021 [30]	3	1	3	7
Vora S.R. et al., 2021 [31]	4	2	2	8
Hancock S. et al., 2024 [32]	4	2	2	8
Verdecchia A. et al., 2024 [33]	4	1	2	7

## Data Availability

The data presented in this study are available in the article.
